# Paediatric Laryngeal Synovial Sarcoma: Dilemmas and Decision-Making

**DOI:** 10.1155/2024/7574240

**Published:** 2024-04-01

**Authors:** Gregory Shein, Alison J. Potter, Christine Loo, Robert Smee, Ian Jacobson, Antoinette Anazodo

**Affiliations:** ^1^Department of Otolaryngology, Sydney Children's Hospital, Randwick, Sydney, NSW 2031, Australia; ^2^Melanoma Institute Australia, Sydney, Australia; ^3^Faculty of Medicine, University of Sydney, Sydney, Australia; ^4^Tissue Pathology and Diagnostic Oncology, Royal Prince Alfred Hospital, Sydney, Australia; ^5^Faculty of Medicine, University of New South Wales, Sydney, Australia; ^6^Department of Anatomical Pathology, Prince of Wales Hospital, Randwick, Sydney, NSW 2031, Australia; ^7^Department of Radiation Oncology, Sydney Children's Hospital, Randwick, Sydney, NSW 2031, Australia; ^8^The UNSW Clinical Teaching School, Prince of Wales Hospital, Sydney, New South Wales, Australia; ^9^Department of Radiation Oncology, Tamworth Base Hospital, Tamworth, New South Wales, Australia; ^10^Department of Otolaryngology, Prince of Wales Hospital, Randwick, Sydney, NSW 2031, Australia; ^11^Kids Cancer Centre, Sydney Children's Hospital, Sydney, NSW 2031, Australia; ^12^Nelune Cancer Centre, Prince of Wales Hospital, Sydney, NSW 2031, Australia; ^13^School of Women's and Children's Health, University of New South Wales, Sydney, NSW 2031, Australia

## Abstract

Primary laryngeal synovial sarcoma is a rare head and neck cancer. We describe a case of synovial sarcoma of the larynx in a previously well 9-year-old boy with a one-month history of a progressively enlarging neck lump. He was referred to our institution after incomplete surgical excision of the then undifferentiated neck mass. A partial laryngectomy including wide local excision of the residual mass was performed. An ipsilateral level I-III neck dissection was also performed concurrently. Clear re-excision margins were achieved. The neck nodes were all negative for metastatic disease. Adjuvant local radiotherapy treatment was administered to reduce the probability of local recurrence. Four years following treatment completion, the patient remains in remission with no signs of treatment-related morbidity. A review of the published literature on laryngeal synovial sarcoma was undertaken. This case represents the youngest patient to be diagnosed with the condition. Surgical excision represents the mainstay of treatment of laryngeal synovial sarcoma. At more common sites of disease, adjuvant radiotherapy has been associated with lower rates of recurrence. However, there is the potential for significant morbidity from irradiating the neck of a paediatric patient. This case report explores the challenges in treating young patients with aggressive head and neck cancers when faced with little available evidence to guide decision-making.

## 1. Introduction

Synovial sarcoma is an uncommon type of soft tissue tumour originating from mesenchymal tissue. Primary occurrences of synovial sarcomas in the head and neck are particularly rare, constituting less than 5% of all synovial sarcomas. Those arising from the larynx are even more uncommon. The case of laryngeal synovial sarcoma typically manifests as a painless lump in the neck. Advanced cases may exhibit airway symptoms such as dysphonia, dyspnea, and stridor. Paediatric cases are exceptionally rare. Diagnosis relies on analysing tumour histopathology, immunohistochemistry, and molecular studies on the resected specimen. The primary treatment for synovial sarcoma is wide local tumour resection. Patients are also typically treated with adjuvant radiotherapy and, less commonly chemotherapy, to reduce the known high rate of recurrence. We describe the case of laryngeal synovial sarcoma in a 9-year-old boy managed at our institution. The challenges in balancing aggressive tumour treatment while attempting to preserve our young patient's laryngeal function are presented.

## 2. Case Presentation

**Figure 1:**
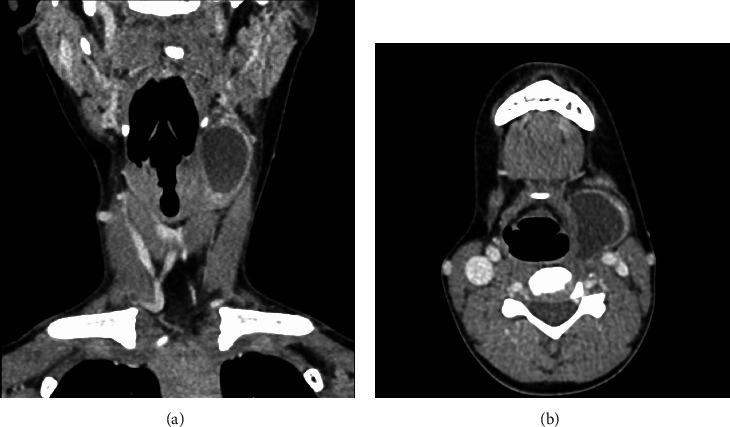
(a) Coronal and (b) axial images of the left-sided neck lump at the time of diagnosis. It measures 2.3 × 3.1 × 4.0 cm.

In May 2017, a 9-year-old boy presented to a local paediatric surgeon with a one-month history of a painless neck lump with no associated symptoms of airway compromise. As part of the assessment, an ultrasound and CT neck were performed. The latter showed a nonspecific 2.3 × 3.1 × 4.0 cm cystic lesion with solid components in the left submandibular fossa (see Figure 1). There was no radiologic evidence of soft tissue or osseous invasion and no adjacent cervical lymphadenopathy evident. Differential diagnoses raised included a branchial cleft cyst, malignancy, or atypical infection. At the time of attempted excision by the paediatric surgeon, the lesion was found to be adherent to the left lateral aspect of the thyroid cartilage, and a decision was made to perform only a partial excision.

**Figure 2:**
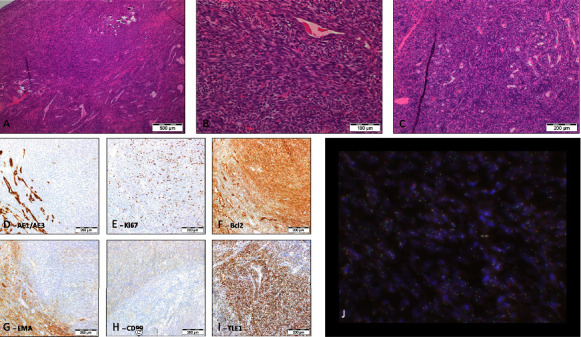
Histopathology and immunochemistry analysis of surgical specimen. (A–C) Histologic appearance of tumour, with biphasic morphology, (D–I) immunohistochemical profile, and (J) FISH^*∗*^ SS18 dual colour break apart rearrangement probe (Vysis).

A microscopic study of the specimen showed a unilocular cyst with nodular proliferation of spindle cells forming irregular intersecting fascicles. Up to 7 mitotic figures were seen per 10 high power fields. There was an invasion into adjacent skeletal muscle and fibrous tissue. Immunoperoxidase stains showed glandular structures and some spindle cells expressing pan keratin (AE1/3). There was a widespread expression of EMA and diffused strong expression of Bcl-2 antigen. A patchy expression of SMA was noted. The Ki67 was estimated to be positive up to 20% of nuclei. There was no expression of ALK-1 antigen and CD21, CD34, CD31, and desmin antigens. Interphase fluorescence in situ hybridisation (FISH) showed a signal pattern consistent with SS18 rearrangement, confirming the diagnosis of a biphasic synovial sarcoma (Figure 2).

**Figure 3:**
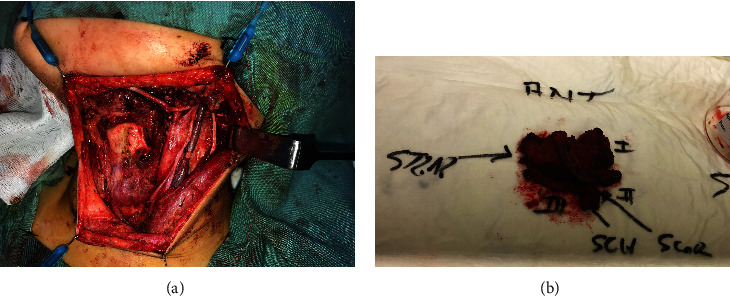
(a) The surgical field at the completion of the partial laryngectomy and left I-IV neck dissection, showing preservation of cranial nerves XI and XII. (b) The resected surgical specimen.

The patient was promptly referred to our multidisciplinary paediatric head and neck cancer service in June 2017. He underwent a staging PET and CT chest, abdomen, and pelvis which showed localised disease with no evidence of metastases. A month following his initial surgery, the tumour was definitively resected by removing the ipsilateral strap muscles and partial laryngectomy, with the removal of the involved left thyroid lamina. An elective left-level I-IV neck dissection was also performed (see Figure 3), and a covering tracheostomy was placed. The left recurrent laryngeal nerve and all lower cranial nerves were preserved. Histopathology confirmed that the residual tumour was completely excised, with the closest margin being 1.3 mm. There was no evidence of tumour in the twenty lymph nodes sampled. He was decannulated 10 days after surgery.

After a review of the literature and following extensive discussions between team members, the patient, and his family, the decision was made to treat with adjuvant intensity-modulated radiation therapy (IMRT). The first fraction was administered 6 weeks following surgery. Given the patient's age, the uninvolved right neck was treated along with the left side to ensure a greater likelihood of neck symmetry in the future. Both right and left anterior neck regions were treated with 2208 cGy in 12 fractions, and the left mid-neck was treated with 2392 cGy in 13 fractions. The radiotherapy total dose was 4600 cGy in 25 fractions. The post-treatment clinical course was complicated by the onset of asymptomatic radiotherapy-induced hypothyroidism identified four months following the completion of radiotherapy treatment. He was referred to a paediatric endocrinologist and commenced regular thyroid hormone supplementation with good effect.

At follow-up six years after the completion of all treatment, he has no dysphonia and no swallowing or breathing concerns. He has mild, intermittent facial swelling that is well-managed with a compression garment. He remains disease-free, with no clinical or radiologic signs of recurrence.

## 3. Discussion

This is the youngest case of laryngeal synovial sarcoma reported in the literature. Only eight other paediatric patients with this condition have been reported to date, with the youngest of those being 11 years old at the time of diagnosis (see Table 1).

In our review of the English-language literature [1], we identified a total of 39 previously reported cases of laryngeal synovial sarcoma. The mean age at diagnosis was 32 years (range 11–79 years) with a male preponderance (69.2%). In 32/39 cases (82.1%), the patient was less than 50 years of age at diagnosis in keeping with the general epidemiology of synovial sarcoma (Fisher reference).

Diagnosis of a head and neck synovial sarcoma is challenging. Several benign entities may behave like synovial sarcoma or show similar radiological features as was apparent in this case. Initial misdiagnoses reported include hemangiopericytoma, branchial cleft cyst, and thyroglossal duct cyst. Where there is diagnostic uncertainty based on imaging studies, we feel that an ultrasound-guided biopsy should be considered. This may have avoided subtotal resection of the tumour that occurred prior to the patient being referred to our institution.

Management of rare paediatric head and neck cancer such as laryngeal synovial sarcoma requires a multidisciplinary, patient-centred approach. In addition to the medical teams, allied health services including speech pathology, physiotherapy, dietetics, psychology, and social workers were all heavily involved in this case from the outset.

Initial management of localised laryngeal synovial sarcoma is predicated on wide local excision of the tumour. Ideally, there should be a margin of 10 mm around the mass, without causing undue functional morbidity from the excision of vital head and neck structures [2]. This often necessitates the need for either partial (as in this case) or complete laryngectomy, with long-term implications for the patient's voice and swallow and social interaction. Preserving native laryngeal function in our paediatric patient was a strong priority but not at the expense of disease removal. A prophylactic ipsilateral neck dissection was performed in our case given the size of the primary tumour and its high metastatic potential, and to ensure disease control in our young patient. Further research is required to determine the indications for a prophylactic neck dissection for head and neck synovial sarcoma.

Our decision to administer radiotherapy to our young patient, who had clear margins and no involved lymph nodes or metastatic disease, was a particularly difficult one. Irradiating the head and neck of paediatric patients has been associated with long-term side effects including trismus, delayed facial bone growth, impaired dentition, xerostomia, and hypothyroidism (as occurred in this case) [3]. Traditionally head and neck sarcoma resections have been associated with high rates of positive margins of up to 42% [4] following surgery. Radiotherapy has thus been used as an adjunct to surgery to achieve locoregional disease control. In their review of head and neck synovial sarcomas, Harb et al. [5] found radiotherapy to be associated with lower recurrence rates and higher survival, although the results did not achieve significance. In our review, we found there was a relatively high rate of recurrence for laryngeal synovial sarcoma 12.8% (5/39) at a mean follow-up period of 3.2 years (range 0.3–16 years). On balance, we felt that achieving disease control for our patient outweighed the short- and long-term morbidity associated with radiotherapy. We noted that among the eight other paediatric patients with laryngeal synovial sarcoma, 7/8 (87.5%) had radiotherapy as part of their treatment regimen (see Table 1) [1]. This rate was significantly higher than the 46.9% (15/32) of adult patients with laryngeal synovial sarcoma treated with radiotherapy as part of their initial treatment regimen [1]. It suggests that, if anything, younger patients tend to be managed more aggressively to achieve disease control.

The role of both neoadjuvant and adjuvant chemotherapy in the management of paediatric head and neck synovial sarcoma is similarly uncertain. Much of the available literature is based on studies involving only adult patients. While early studies suggested adjuvant chemotherapy confers no survival benefit, the Sarcoma Meta-Analysis Collaboration found adjuvant chemotherapy improved time to local recurrence and distant recurrence and overall recurrence-free survival among adult patients with localised resectable disease [6]. In 4/8 cases of paediatric laryngeal synovial sarcoma reported in the literature, chemotherapy was used as part of the treatment regimen. In our case, it was decided that chemotherapy not be administered given the localised disease and absence of adverse features on histology. The role of chemotherapy where disease control is not achieved through surgery requires further research.

## 4. Conclusion

The perennial challenges in diagnosing synovial sarcomas are evident in this case. The case also highlights the challenges in treating young patients with rare and aggressive head and neck cancers with limited evidence to guide decision-making. Our case shows that in spite of these obstacles, disease-free long-term survival with excellent laryngeal function can be achieved. Improvements in our understanding of the indications for adjuvant radiotherapy and chemotherapy may allow for a more targeted treatment approach for paediatric patients without compromising cure rates.

## Figures and Tables

**Table 1:** Paediatric cases of laryngeal synovial sarcoma (*n* = 9).

Author/Year	Country of treatment	Age/Sex	Laryngeal sites	Maximal diameter (cm)	Subtype	Management^*∗*^	Follow-up (years)	Status at follow-up
Shein 2021 (Current case)	Australia	9M	L lateral aspect of thyroid cartilage	4.0	Biphasic	Wide local excision, selective neck dissection, partial laryngectomy, and + RTX	1.0	NED
Alhatem 2019	USA	10M	Left supraglottis, aryepiglottic fold		Monophasic	Embolization, I, RTX, and total laryngectomy	15	Not recorded
Simon 2012	UK	11M	R aryepiglottic fold		Not recorded	I, DOX, RTX (50.4 Gy), total laryngectomy, and thyroidectomy		NED
Fernández-Aceñero 2009	Spain	12M	Supraglottic		Not recorded	Wide local excision and chemotherapy	0.33	NED
Feng 2017	China	14M	L aryepiglottic fold	8	Biphasic	Wide surgical excision, partial laryngectomy, and RTX	1.5	NED
Morland 1994	UK	14M	Arytenoids		Biphasic	Debulking, I, ACD, V, RTX (60 Gy), and total laryngectomy	0.83	NED
Papaspyrou 2003	Greece	16M	Aryepiglottic folds and hypopharyngeal walls	4	Biphasic	Endoscopic CO2 laser resection and RTX (50 Gy)	2	NED
Javed 2015	Pakistan	16F	Supraglottis	5	Not recorded	Lateral pharyngectomy, wide local excision, and RTX (60 Gy)	2	NED
Reddy 2015	India	17F	L supraglottis, arising from L aryepiglottic fold		Not recorded	Endoscopic resection and RTX (44 Gy)		NED

^∗^Management listed in order of treatments administered. Left, L; right, R; RTX, radiotherapy; I, Ifosfamide; CIS, cisplatin, CYC, cyclophosphamide, V, vincristine, DOX, doxorubicin; ACD, actinomycin D; docetaxel, DOC; NED, no evidence of disease.

## Data Availability

The data used to support the findings of this study are available upon request.
